# Versatile kinematics-based constraint identification applied to robot task reproduction

**DOI:** 10.3389/frobt.2025.1574110

**Published:** 2025-07-15

**Authors:** Alex H. G. Overbeek, Douwe Dresscher, Herman van der Kooij, Mark Vlutters

**Affiliations:** ^1^ Department of Biomechanical Engineering, University of Twente, Enschede, Netherlands; ^2^ Department of Robotics and Mechatronics, University of Twente, Enschede, Netherlands

**Keywords:** constraint identification, physical constraints, constraint frames, contact modeling, robot manipulation, learning from demonstration, imitation learning

## Abstract

Identifying kinematic constraints between a robot and its environment can improve autonomous task execution, for example, in Learning from Demonstration. Constraint identification methods in the literature often require specific prior constraint models, geometry or noise estimates, or force measurements. Because such specific prior information or measurements are not always available, we propose a versatile kinematics-only method. We identify constraints using constraint reference frames, which are attached to a robot or ground body and may have zero-velocity constraints along their axes. Given measured kinematics, constraint frames are identified by minimizing a norm on the Cartesian components of the velocities expressed in that frame. Thereby, a minimal representation of the velocities is found, which represent the zero-velocity constraints we aim to find. In simulation experiments, we identified the geometry (position and orientation) of twelve different constraints including articulated contacts, polyhedral contacts, and contour following contacts. Accuracy was found to decrease linearly with sensor noise. In robot experiments, we identified constraint frames in various tasks and used them for task reproduction. Reproduction performance was similar when using our constraint identification method compared to methods from the literature. Our method can be applied to a large variety of robots in environments without prior constraint information, such as in everyday robot settings.

## 1 Introduction

Autonomous robotic manipulation has the potential to improve human lives by alleviating physical effort. Robots may offer advantages over human labor regarding consistency, endurance, strength, accuracy, and/or precision in fields such as healthcare, logistics, exploration, and the manufacturing industry. However, autonomous robots are typically designed for one specific task in one specific environment, hence they lack versatility. Tasks in different environments therefore often require different robots, which may be expensive and impractical.

It may therefore be beneficial to develop robots that are versatile in their task execution, allowing a single robot to deal with a variety of tasks and environments. Manipulation tasks may include pick-and-place tasks (e.g., order picking), contact tasks (e.g., wiping, polishing, contour following, and opening/closing compartments) and tool-use tasks (e.g., hammering and screwing). Common environments may include moveable objects such as tools, as well as (immovable) physical constraints.

Models of the physical environments may assist in versatile task execution. For example, door opening is a common everyday task involving similar articulation mechanisms across most doors. If the interaction mechanisms (e.g., hinges and slides) can be modeled, a robot may apply the same control to all environments that have the same mechanisms, thereby improving robot versatility. However, manually creating such models may be cumbersome due to many possible variations in the environment, such as object positions, orientations, shapes, and dynamics. There is therefore a need for automatic modeling of physical environments (Kroemer et al., 2020).

Automatically modeling physical environments may occur in a Learning from Demonstration (LfD) context (Kroemer et al., 2020). In LfD, robots learn to perform tasks from human demonstrations, for example, by manually guiding a robot, rather than by explicit programming. From the demonstration data, the physical interactions throughout the demonstration may be modelled, which in turn may be used in autonomous task reproduction.

Physical environments typically contain kinematic constraints that restrict movement. In everyday settings numerous constraints may be encountered, including articulated/mechanism contacts, such as prismatic (drawers) and revolute joints (doors), polyhedral contacts such as pin-plane contacts (pen drawing) and plane-plane contact (box sliding), and contour-following contacts (dusting).

Constraint awareness can benefit task execution in several ways. First, tasks can often be simplified when expressed in the constraints (Bruyninckx and De Schutter, 1996). Second, choosing a suitable control method, e.g., position, velocity, force, or impedance control can improve both task performance and stability by keeping undesirable interaction forces low (Conkey and Hermans, 2019; De Schutter et al., 1999). Third, planning to avoid constraints can simplify some tasks because there are fewer state transitions to consider, such as in reaching tasks (Oriolo and Vendittelli, 2009). Alternatively, planning to introduce constraints can simplify some tasks by reducing the free space of the robot, for example, during object alignment (Suomalainen et al., 2021). Fourth, differentiating between constrained states in a task provides meaningful, tractable building blocks, which can improve task planning (Ureche et al., 2015; Jain and Niekum, 2018; Holladay et al., 2021) and facilitate learning (Simonič et al., 2024). Fifth, some constraints are common in many robot tasks and can therefore be a basis for generalization between tasks (Li and Brock, 2022; Li et al., 2023).

Kinematic constraints consist of a (i) constraint class, e.g., a type of joint or contact, and (ii) constraint geometry, e.g., the orientation and position of a rotation axis or surface normal (De Schutter et al., 1999). Identifying constraints from data therefore requires (i) classifying the constraint class and (ii) identifying the constraint geometry, both are the topic of this work.

In this work, we make three assumptions about robot manipulation to limit our scope. First, we assume that robots are rigidly attached to a constrained mechanism or object in contact with the environment, and thus leave grasping and environmental compliance out of our scope. Second, we assume that there is a single maintained contact between a robot and the environment, such as a contact point, line, plane, or other continuous contact area. Third, we assume that all unconstrained degrees of freedom are excited, to prevent ambiguity between true physical constraints that do not allow motion and non-observed motions.

Bruyninckx and De Schutter defined kinematic constraint classes based on contact (Bruyninckx and De Schutter, 1993a; Bruyninckx et al., 1993b). They proposed to identify constraints by two methods: first, based on the absence of mechanical power in the direction of constraints, requiring position and force measurements. Second, based on whether velocities or forces separately fit candidate constraint models. They used both methods in a Kalman filter with a candidate constraint model of a specific class to estimate constraint geometry, such as contact points, axes of rotations, and polyhedral contact normals (De Schutter et al., 1999).

Several authors extended the methods of Bruyninckx and De Schutter, mainly on multi-contact polyhedral contacts (Meeussen et al., 2007; Cabras et al., 2010; Lefebvre et al., 2005). They identify, classify, and segment data containing arbitrary contacts between two uncertain polyhedral or curved objects using pose and wrench measurements. Where previous methods required approximate geometric models of the polyhedra, Slaets et al. identify arbitrary unknown polyhedra at runtime (Slaets et al., 2007). Although polyhedrons are useful to approximate many tasks, these methods are fundamentally limited in modeling rotations.

Alternative methods omit noise models, and fit constraint models of a specific class directly to data, identifying the constraint geometry in the process. Such models are specified manually, and when several candidate models are proposed, the best-fitting model is chosen. For example, several authors identify one of three candidate models (fixed, prismatic, and revolute joints) using kinematic measurements (Sturm et al., 2010; Niekum et al., 2015; Hausman et al., 2015). Subramani et al. identify six candidate models using kinematic measurements (Subramani et al., 2018). They later expand their method with force information and identify eight candidate models (Subramani et al., 2020).

Some methods do not require specific constraint models but use more versatile representations to capture multiple constraints. Sturm et al. fit Gaussian processes in configuration space, but the identified Gaussian process kernel parameters are not straightforward to interpret (Sturm et al., 2011). Mousavi Mohammadi et al. identify “task frames” instead of identifying constraints explicitly (Mousavi Mohammadi et al., 2024). Such frames conveniently describe contact tasks and thereby often align with constraint geometry, but lack constraint classification (Bruyninckx and De Schutter, 1996). They use velocities and/or forces in a multi-step decision process to identify frame properties that result in low or constant velocities and forces with minimal uncertainty. Van der Walt et al. identify constraints from kinematic data by fitting points, lines, planes, and their higher dimensional equivalents in six dimensional linear and angular velocity space, after which they select the best fitting model (Van der Walt et al., 2025).

Because kinematic constraints occur often in robot manipulation tasks, we believe more versatile constraint identification is an important step to advance the applicability of robots in the real world. However, the literature on constraint identification shows two common limitations. First, methods in the literature often require specific prior constraint models and estimates of geometry and noise. In everyday robot settings where many constraints can be encountered, it may be challenging to exhaustively define such models and estimates. Second, various methods require force measurements, which may not be available. This work overcomes both limitations by identifying *constraint frames* from kinematic data. First, the method can identify a wide variety of constraints without specific prior constraint models, or estimates of geometry or noise. The method is used to identify articulated/mechanism contacts, polyhedral contacts, and contour following contacts. Second, the method only requires kinematic measurements. Therefore, our method can be applied to various robots in everyday settings, without prior information about the environment or task.

The method offers two more useful features. First, the identified constraint frames can be used directly for task reproduction since the constraints are expressed in a task-relevant reference frame. Second, the number of identified parameters is fixed, in contrast to methods that use an increasing number of parameters for each added candidate model.

This paper is organized as follows: Section 2 discusses preliminaries on rigid body kinematics. Section 3 proposes to specify constraints using constraint frames. Section 4 proposes an optimization problem to identify constraint frames from kinematic data. Section 5 evaluates the method on experimental data from simulation and real robot task demonstrations. Section 6 reproduces robot tasks using the identified constraint frames. Section 7 discusses the results and draws conclusions.

## 2 Preliminaries on kinematics

This section contains preliminaries for rigid bodies kinematics (Lynch and Park, 2017). Motion between two rigid bodies can be represented by the transformation between two reference frames, one frame rigidly attached to each body. Such reference frames have a position and orientation in space, which can change with body motion. The transformation between two reference frames in three-dimensional space can be parameterized by a distance between their positions $p\in R^{3}$ and a rotation matrix $R\in SO\left(3\right)$ between their orientations. These components can be combined in a homogenous transformation matrix

$$T=\left[\begin{array}{cc} R & p\\ 0_{1\times 3} & 1 \end{array}\right]\in SE\left(3\right),$$

which represents the pose (orientation R and position p ) of one frame relative to the other.

The velocity between two rigid bodies can be represented by a twist $V={\left[\omega^{T},v^{T}\right]}^{T}\in R^{6}$ , which contains an angular velocity $\omega \in R^{3}$ and a linear velocity $v\in R^{3}$ . The twist must be expressed in a reference frame, denoted by a superscript, e.g., $V^{b}={\left[{\left(\omega^{b}\right)}^{T},{\left(v^{b}\right)}^{T}\right]}^{T}$ for reference frame $\left\{b\right\}$ . If T describes the pose of a body frame $\left\{b\right\}$ with respect to a ground frame $\left\{g\right\}$ , the twist $V^{b}$ of a body with respect to the ground expressed in frame $\left\{b\right\}$ is related to T by:

$$T^{-1}\dot{T}={\left[V^{b}\right]}_{\times }=\left[\begin{array}{cc} {\left[\omega^{b}\right]}_{\times } & v^{b}\\ 0_{1\times 3} & 0 \end{array}\right]\in se\left(3\right),$$ (1)

where ${\left[\omega \right]}_{\times }$ is the skew-symmetric representation of $\omega ={\left[\omega_{1},\omega_{2},\omega_{3}\right]}^{T}$ :

$${\left[\omega \right]}_{\times }=\left[\begin{array}{ccc} 0 & -\omega_{3} & \omega_{2}\\ \omega_{3} & 0 & -\omega_{1}\\ -\omega_{2} & \omega_{1} & 0 \end{array}\right]\in so\left(3\right).$$

Expressing the twist $V^{b}$ in a frame other than $\left\{b\right\}$ requires the pose matrix of $\left\{b\right\}$ with respect to the new frame. For example, if R and p again represent the pose of body frame $\left\{b\right\}$ with respect to the ground frame $\left\{g\right\}$ , the transformation

$$V^{g}=\left[\begin{array}{cc} R & 0_{3\times 3}\\ {\left[p\right]}_{\times }R & R \end{array}\right]V^{b}.$$ (2)

expresses the twist $V^{b}$ in frame $\left\{g\right\}$ .

## 3 Constraint frames

This section introduces a method to specify constraints that result in constrained kinematics. This method is used in Section 4 for the inverse problem: identifying constraints from kinematic data.

### 3.1 Degrees of freedom

Kinematic constraints limit a robot’s motion. We consider Pfaffian constraints on the end effector twist V of a robot with respect to the ground. The form of such constraints is

$$A\left(T\right)V=0_{h},$$ (3)

where $A\left(T\right)\in R^{h\times 6}$ defines h≤6 constraint equations (Lynch and Park, 2017).

We restrict $A\left(T\right)$ to be coordinate transformations of the form of Equation 2 that transform velocities ω and/or v to *one or more* axes of some *to be determined* frame $\left\{\xi \right\}$ . For example, the i -th constraint equation $A_{i}\left(T\right)$ may restrict the j -th axis of ω when expressed in $\left\{\xi \right\}$ , such that $A_{i}\left(T\right)V=\omega_{j}^{\xi }=0.$ Constraints can similarly be applied to v .

We also allow constraints on ω and v to be described from different frames, respectively. We denote the frame for ω as $\left\{\xi \right\}=\left\{\phi \right\}$ and for v as $\left\{\xi \right\}=\left\{\psi \right\}$ . Therefore, we consider constraints of the form:

$$\begin{array}{c} \omega_{n}^{\phi }=0,\\ v_{m}^{\psi }=0. \end{array}$$ (4)

Here, axis n of ω is constrained in $\left\{\phi \right\}$ , and axis m of v is constrained in $\left\{\psi \right\}$ . For either velocity, the number of constraints can be none, one, two, or three. We thus consider *bilateral zero-velocity constraints along one or more axes of* ω *expressed in* $\left\{\phi \right\}$ *and* v *expressed in* $\left\{\psi \right\}$ . Although most contact constraints are unilateral, they can be modeled as bilateral if contact is kept, which we assume in this work.

To specify whether the axes of $\omega^{\phi }$ are constrained according to (4), we use an associated binary degree of freedom vector $d^{\phi }\in {\left\{0,1\right\}}^{3}$ . Here, 0 indicates the associated axis is constrained and 1 indicates it is unconstrained. The analogous situation applies to v with $d^{\psi }\in {\left\{0,1\right\}}^{3}$ .

By summing the degree of freedom vector $\sum_{n=1}^{3}d_{n}^{\phi }\in \left\{0,1,2,3\right\}$ , we obtain the physical degrees of freedom of ω in frame $\left\{\phi \right\}$ . When adding units, $d^{\phi }\text{ rad }s^{-1}=\text{span}\left(\omega^{\phi }\right)$ gives a basis for ω , which represents all possible ω being either zero, lying on a line, on a plane, or in a volume.

Constraints can be thus defined by specifying $d^{\phi }$ and $d^{\psi }$ . For example, a revolute joint can be specified by $d^{\phi }={\left[1,0,0\right]}^{T}$ if the first axis of $\left\{\phi \right\}$ aligns with the rotation axis, and $d^{\psi }={\left[0,0,0\right]}^{T}$ if the position of $\left\{\psi \right\}$ is on the rotation axis.

### 3.2 Frame type and geometry

So far, the frame $\left\{\phi \right\}$ in which to express constraints on ω , and frame $\left\{\psi \right\}$ in which to express constraints on v , have not been specified. Here, we require the frames to be attached to the ground body G *or* the robot body B , similar to Mousavi Mohammadi et al. (2024).

**FIGURE 1:**
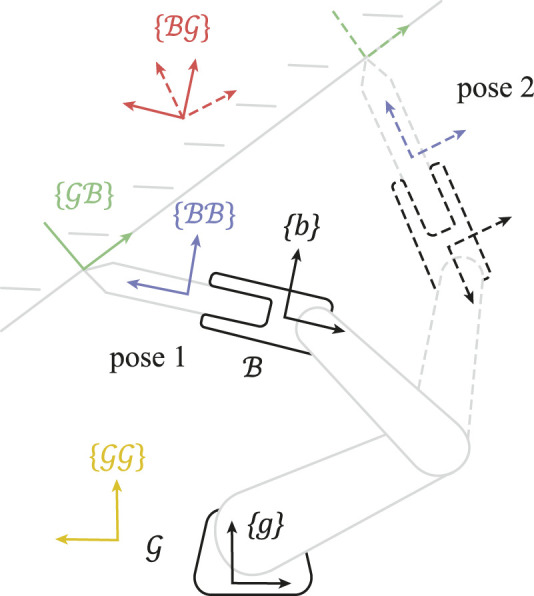
Constraint frame identification following a two-dimensional task demonstration, e.g., by a human guiding the robot. The behavior of the constraint frame types $\left\{\psi \right\}\in \left\{\left\{GG\right\},\left\{GB\right\},\left\{BG\right\},\left\{BB\right\}\right\}$ is shown at two instants (solid and dashed) during a demonstration of robot body B with respect to ground body G . Frames $\left\{GG\right\}$ and $\left\{BB\right\}$ are rigidly attached to their respective bodies. Frames $\left\{GB\right\}$ and $\left\{BG\right\}$ have constant orientation in one body, but constant position in the other. Only the transformations between ground $\left\{g\right\}$ and body $\left\{b\right\}$ are measured (black), and the method identifies the unknown (gray) constraint between the tool and ground. In this example, the optimal frame is $\left\{GB\right\}$ at tip of the tool, because the linear velocities along one of its axes are always zero, which is not the case for velocities expressed in the other frames. In practice, the method performs a continuous optimization in three-dimensional space to find the frame position and orientation, and also identifies the angular velocity constraint frame.

The v frame $\left\{\psi \right\}$ must have its orientation constant in G *or* B , and its position constant in G *or* B . This results in four frame types denoted by $\left\{\psi \right\}\in \left\{\left\{GG\right\},\left\{GB\right\},\left\{BG\right\},\left\{BB\right\}\right\}$ . For example, $\left\{BG\right\}$ indicates that the frame’s orientation is constant in ground body B and its position is constant in body G . Figure 1 visualizes these frame types during motion.

Because the frames’ orientations and positions are constant in either one of the bodies, they can be parameterized by a constant rotation matrix $R^{\psi }$ and position vector $p^{\psi }$ . For example, $R^{BG}$ denotes the constant rotation matrix between $\left\{BG\right\}$ and a frame $\left\{b\right\}$ on body B (Figure 1).

For the ω frame $\left\{\phi \right\}$ , only two types of constraint frames have to be considered because ω is independent of the position of the frame it is expressed in, that is $\omega^{GG}=\omega^{GB}$ and $\omega^{BG}=\omega^{BB}$ . Therefore, the dependence on frame position can be omitted. The two frame types are $\left\{\phi \right\}\in \left\{\left\{G\right\},\left\{B\right\}\right\}$ , which are parameterized solely by a constant rotation matrix $R^{\phi }$ . Because frames $\left\{\phi \right\}$ do not have a position, they are shown at convenient positions in figures.

### 3.3 Applied constraint frames

**FIGURE 2:**
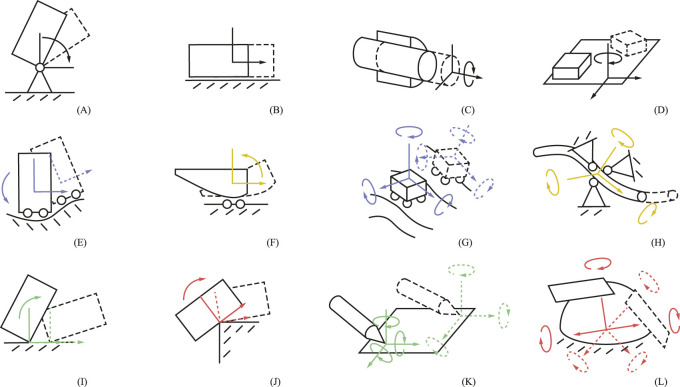
Visualization of twelve (not exhaustive) constraints that can be captured by the method. Each constraint ** (A–L)** is described in Table 2. The left-hand figures are planar, where the rotation axis (y-axis) is normal to the plane. Colors (Table 2) indicate the linear velocity constraint frame type $\left\{\psi \right\},\;\mathrm{where}\;\left\{GG\right\}\;\mathrm{is}\;\mathrm{yellow},\;\left\{GB\right\}\;\mathrm{is}\;\mathrm{green},\;\left\{BG\right\}\;\mathrm{is}\;\mathrm{red},\;\mathrm{and}\;\left\{BB\right\}\;\mathrm{is}\;\mathrm{blue}$ . For conciseness, angular velocity constraint frames $\left\{\phi \right\}$ overlap exactly with the linear velocity constraint frames $\left\{\psi \right\}$ , such that $R^{\phi }=R^{\psi }$ . If a frame at the second time instance (dashed) is not shown, it is the same as the first. The linear velocity degrees of freedom $d^{\psi }$ are marked along the frame axes by an arrowhead (unconstrained) or no arrowhead (constrained). The unconstrained angular velocity degrees of freedom $d^{\phi }$ are marked by a rotational vector.

In summary, degrees of freedom $d^{\phi }$ and $d^{\psi }$ specify whether axes are constrained; types $\left\{\phi \right\}\in \left\{\left\{G\right\},\left\{B\right\}\right\}$ and $\left\{\psi \right\}\in \left\{\left\{GG\right\},\left\{GB\right\},\left\{BG\right\},\left\{BB\right\}\right\}$ specify what the frames are attached to; and $R^{\phi }$ , $R^{\psi }$ , and $p^{\psi }$ specify the geometry of the constraints. The combination of degrees of freedom and frame types can be considered the *class* of a constraint. Table 1 summarizes these properties and the constant parameters that specify them. Figure 2 and Table 2 show twelve (not exhaustive) example constraints that can be specified using this method.

**TABLE 1:** Parameters of the proposed method.

Velocity $R^{3}$	Constraint frame			
	DOF	Type	Geometry	
	Binary ${\left\{0,1\right\}}^{3}$	$\left\{const.\;pos.\;vector\;in,\right.$ $\left.const.\;rot.\;matrix\;in\right\}$	Rot. matrix $SO\left(3\right)$	Pos. vector $R^{3}$
ω (angular)	$d^{\phi }$	$\left\{\phi \right\}\in \left\{\left\{G\right\},\left\{B\right\}\right\}$	$R^{\phi }$	−
v (linear)	$d^{\psi }$	$\left\{\psi \right\}\in \left\{\left\{BB\right\},\left\{GB\right\},\left\{GG\right\},\left\{BG\right\}\right\}$	$R^{\psi }$	$p^{\psi }$

The presented method does not uniquely define constraints for three reasons. First, the ordering of the axes is undefined. For example, $d={\left[1,0,0\right]}^{T}$ and $R=\left[r_{1},r_{2},r_{3}\right]$ define the same constraint as $d={\left[0,1,0\right]}^{T}$ and $R=\left[r_{2},r_{1},r_{3}\right]$ . Second, the positive or negative directions of the axes are irrelevant because we consider bilateral constraints. Therefore, the basis vectors in $R=\left[\pm r_{1},\pm r_{2},\pm r_{3}\right]$ can have either sign. Third, (parts of) R are always free to choose, as shown in Table 2.

**TABLE 2:** Parameters of the example constraints of Figure 2.

Figure 2 label	Constraint name	Constraint frame			
		DOF	Type	Geometry	
		$d^{\phi }$	$\left\{\phi \right\}$	$R^{\phi }$	−
		$d^{\psi }$	$\left\{\psi \right\}$	$R^{\psi }$	$p^{\psi }$
A	Revolute joint	$\left[0,1,0\right]$	−	$\left[-,r_{y},-\right]$	−
		$\left[0,0,0\right]$	−	$\left[-,-,-\right]$	$\left[p_{x},-,p_{z}\right]$
B	Prismatic joint	$\left[0,0,0\right]$	−	$\left[-,-,-\right]$	−
		$\left[1,0,0\right]$	−	$\left[r_{x},-,-\right]$	$\left[-,-,-\right]$
C	Cylinder joint	$\left[1,0,0\right]$	−	$\left[r_{x},-,-\right]$	−
		$\left[1,0,0\right]$	−	$\left[r_{x},-,-\right]$	$\left[-,p_{y},p_{z}\right]$
D	Plane-plane contact	$\left[0,0,1\right]$	−	$\left[-,-,r_{z}\right]$	−
		$\left[1,1,0\right]$	−	$\left[-,-,r_{z}\right]$	$\left[-,-,-\right]$
E	Planar contour following	$\left[0,1,0\right]$	−	$\left[-,r_{y},-\right]$	−
		$\left[1,0,0\right]$	$\left\{BB\right\}$	$\left[r_{x},-,-\right]$	$\left[p_{x},-,-\right]$
F	Planar contour rolling	$\left[0,1,0\right]$	−	$\left[-,r_{y},-\right]$	−
		$\left[1,0,0\right]$	$\left\{GG\right\}$	$\left[r_{x},-,-\right]$	$\left[p_{x},-,-\right]$
G	Cart on a curved surface	$\left[1,1,1\right]$	−	$\left[-,-,-\right]$	−
		$\left[1,1,0\right]$	$\left\{BB\right\}$	$\left[-,-,r_{z}\right]$	$\left[p_{x},p_{y},p_{z}\right]$
H	Contour rolling	$\left[1,1,1\right]$	−	$\left[-,-,-\right]$	−
		$\left[1,0,0\right]$	$\left\{GG\right\}$	$\left[r_{x},-,-\right]$	$\left[p_{x},p_{y},p_{z}\right]$
I	Planar pin-plane contact	$\left[0,1,0\right]$	−	$\left[-,r_{y},-\right]$	−
		$\left[1,0,0\right]$	$\left\{GB\right\}$	$\left[r_{x},-,-\right]$	$\left[p_{x},-,p_{z}\right]$
J	Planar plane-pin contact	$\left[0,1,0\right]$	−	$\left[-,r_{y},-\right]$	−
		$\left[1,0,0\right]$	$\left\{BG\right\}$	$\left[r_{x},-,-\right]$	$\left[p_{x},-,p_{z}\right]$
K	Pin-plane contact	$\left[1,1,1\right]$	−	$\left[-,-,-\right]$	−
		$\left[1,1,0\right]$	$\left\{GB\right\}$	$\left[-,-,r_{z}\right]$	$\left[p_{x},p_{y},p_{z}\right]$
L	Plane-pin contact	$\left[1,1,1\right]$	−	$\left[-,-,-\right]$	−
		$\left[1,1,0\right]$	$\left\{BG\right\}$	$\left[-,-,r_{z}\right]$	$\left[p_{x},p_{y},p_{z}\right]$

“ − ” means that entry is not uniquely defined.

In addition, two types of redundancies can occur depending on the constraint. First, (parts of) the frame $\left\{\psi \right\}$ positions $p^{\psi }$ may be free to choose. For example, the frame $\left\{\psi \right\}$ of a cylinder joint (Figure 2C) may lie anywhere on its rotation axis. Second, multiple frame types may lead to the same constraints. For example, any of the four frame types of $\left\{\psi \right\}$ can be used to specify a revolute joint (Figure 2A).

For simplicity, we consider angular velocity constraints where either type $\left\{\phi \right\}\in \left\{\left\{G\right\},\left\{B\right\}\right\}$ leads to the same constraint, i.e., when $\sum_{n=1}^{3}d_{n}^{\phi }\neq 2$ .

## 4 Constraint frame identification

Section 3 introduced a method to specify constraints that result in constrained kinematics. This section considers the inverse problem: identifying constraints from constrained kinematic data. Constraint identification is first defined as an optimization problem for angular velocity ω , followed by a second optimization problem for linear velocity v .

Section 3 specified constraints on ω through a constraint frame $\left\{\phi \right\}$ with a degree of freedom vector $d^{\phi }$ . The inverse problem, given measured ω , is to find *some* $\left\{\phi \right\}$ that results in *some* $d^{\phi }$ . To resolve this ambiguity, we aim to find the $\left\{\phi \right\}$ in which $\omega^{\phi }$ has the minimum number of degrees of freedom and thus the maximum number of constraints. Thus, we define our constraint frame identification as:

$$\min_{\phi }\sum_{n=1}^{3}d_{n}^{\phi }\in \left\{0,\ldots ,3\right\}.$$ (5)

Thereby, a minimal representation of ω is found by expressing it as $\omega^{\phi }$ .

Measuring ω at samples $k\in \left\{1,2,\ldots ,K\right\}$ yields a 3×K matrix Ω with rows $\omega_{n}$ , columns $\omega \left(k\right)$ , and entries $\omega_{n}\left(k\right)$ . If the constraint condition of Equation 4 holds for an axis n , then $\omega_{n}^{\phi }\left(k\right)=0$ should hold for all samples k . To assess the velocity magnitude in measurement data we use the scaled 2 -norm, or root mean square (RMS), over all samples of an axis n :

$$\lambda_{n}^{\phi }={\left\|\omega_{n}^{\phi }\right\|}_{2},$$

as defined by the scaled p -norm with p=2 :

$${\left\|x\right\|}_{p}={\left(\frac{1}{K}\sum_{k=1}^{K}{\left|x\left(k\right)\right|}^{p}\right)}^{\frac{1}{p}},$$

for a signal x with samples $x\left(k\right)$ (Bullen, 2003).

With noiseless measurements, the constraint condition (Equation 4) can then be tested by $d_{n}^{\phi }=0$ if $\lambda_{n}^{\phi }=0$ and $d_{n}^{\phi }=1$ if $\lambda_{n}^{\phi }>0$ . When noise is present however, noise is added to the zero-velocity constraint and the constraint condition (Equation 4) becomes

$$\omega_{n}^{\phi }\left(k\right)\;∼\;N\left(0,\sigma^{2}\right),$$

assuming normally distributed noise N with variance $\sigma^{2}$ . Therefore, the norm $\lambda_{n}^{\phi }$ is not bounded from below by 0 , but by the noise variance $\sigma^{2}$ , which is the RMS of a normally distributed discrete signal with zero mean. Because $\lambda_{n}^{\phi }\geq \sigma^{2}>0$ , evaluating $d_{n}^{\phi }$ will always lead to $d_{n}^{\phi }=1$ , and an optimization of Equation 5 has no gradient.

Instead of minimizing over $d_{n}^{\phi }$ , we minimize $\lambda_{n}^{\phi }$ directly as a continuous measure of a signal’s degree of freedom. We aim to find a frame $\left\{\phi \right\}$ in which the variance of each of the axes $\lambda_{n}^{\phi }$ in $\lambda^{\phi }\in R^{3}$ are minimized. The values in $\lambda^{\phi }$ are bounded from below by $\sigma^{2}$ if constraints are present, but are larger otherwise. Because we are interested in finding those constraints, we prefer finding small individual values in $\lambda^{\phi }$ rather than larger values.

Therefore, we use a p -norm over the three axes of $\lambda^{\phi }$ :

$$\min_{\phi }{\left\|\lambda^{\phi }\right\|}_{p},$$

but with −∞<p<1 , for which the minimization is more sensitive to small individual values in $\lambda^{\phi }$ in contrast to p>1 . Some values of p yield special simplified expressions (Bullen, 2003). For example, p=1 yields the mean of $\lambda^{\phi }$ , which is equally sensitive to all values in $\lambda^{\phi }$ regardless of size. The limit p=−∞ yields the minimum of $\lambda^{\phi }$ , which is only sensitive to the smallest value in $\lambda^{\phi }$ . Here we use p=0 , which yields the following simplified expression:

$$\min_{\phi }{\left\|\lambda^{\phi }\right\|}_{p=0}=\min_{\phi }{\left(\prod_{n=1}^{3}\lambda_{n}^{\phi }\right)}^{1/3},$$

also known as the geometric mean (Bullen, 2003). This results in a quasinorm, because not all conditions for a norm are satisfied. By substituting the 2 -norms (RMS) and 0 -quasinorm (geometric mean) into one entry-wise 2,0 -quasinorm, we obtain

$$\min_{\phi }{\left\|\Omega^{\phi }\right\|}_{2,0}=\min_{\phi }{\left(\prod_{n=1}^{3}{\left(\frac{1}{K}\sum_{k=1}^{K}{\left|\omega_{n}^{\phi }\left(k\right)\right|}^{2}\right)}^{\frac{1}{2}}\right)}^{\frac{1}{3}}.$$ (6)

Section 3 proposed two options for $\left\{\phi \right\}\in \left\{\left\{G\right\},\left\{B\right\}\right\}$ , both parameterized by constant $R^{\phi }$ , and thus two optimization problems. The measurement matrix Ω can be expressed in frame $\left\{\phi \right\}$ using the coordinate transformation of Equation 2, yielding

$$\min_{R^{\phi }}{\left\|R^{\phi }\;\Omega \right\|}_{2,0},$$ (7)

where the first optimization $\left.\left\{\phi \right.\}=\{B\right\}$ uses $\Omega =\Omega^{b}$ and the second optimization $\left\{\phi \right\}=\left\{G\right\}$ uses $\Omega =\Omega^{g}$ .

This method can also be applied to linear velocity v , with a measured 3×K matrix V :

$$\min_{\psi }{\left\|V^{\psi }\right\|}_{2,0}.$$

Section 3 proposed four options for $\left\{\psi \right\}\in \left\{\left\{GG\right\},\left\{GB\right\},\left\{BG\right\},\left\{BB\right\}\right\}$ , all parameterized by constant $R^{\psi }$ and $p^{\psi }$ , and thus four optimization problems. The measured V can be expressed in frame $\left\{\psi \right\}$ using the coordinate transformation of Equation 2, yielding

$$\min_{R^{\psi },p^{\psi }}{\left\|R^{\psi }\;\bar{R}\left(\bar{V}+{\left[p^{\psi }\right]}_{\times }\;\bar{\Omega }\right)\right\|}_{2,0},$$ (8)

where $\bar{\Omega }$ , $\bar{V}$ , $\bar{R}$ are given in Table 3, depending on which of the four frame types they are expressed in.

**TABLE 3:** Kinematic variables to transform (Equation 8).

$\left\{\psi \right\}$	$\bar{\Omega }$ from (2)	$\bar{V}$ from (2)	$\bar{R}$
$\left\{BB\right\}$	${\bar{\Omega }}^{b}$	${\bar{V}}^{b}$	$-\left[I_{3\times 3},I_{3\times 3},\ldots \right]$
$\left\{GB\right\}$	${\bar{\Omega }}^{b}$	${\bar{V}}^{b}$	$\left[R\left(0\right),R\left(1\right),\ldots \right]$
$\left\{GG\right\}$	${\bar{\Omega }}^{g}$	${\bar{V}}^{g}$	$-\left[I_{3\times 3},I_{3\times 3},\ldots \right]$
$\left\{BG\right\}$	${\bar{\Omega }}^{g}$	${\bar{V}}^{g}$	$\left[R^{T}\left(0\right),R^{T}\left(1\right),\ldots \right]$

Because the ground-truth constraint frame types $\left\{\phi \right\}$ and $\left\{\psi \right\}$ will not be known beforehand, both optimizations of Equation 7 and all four of Equation 8 must be done. Then, $\left\{\phi \right\}$ and $\left\{\psi \right\}$ can be classified by choosing the type with the lowest minimized quasinorm. Furthermore, the binary degrees of freedom vectors d can be classified by thresholding λ .

The inputs to the method are twists $V\left(k\right)$ containing $\omega \left(k\right)$ in Ω and $v\left(k\right)$ in V , and poses $T\left(k\right)$ containing $R\left(k\right)$ and $p\left(k\right)$ . The outputs are one set of the constraint frame parameters of Table 1: degrees of freedom, type, and geometry.

## 5 Evaluation

To evaluate the identification method, we simulated kinematic data for all twelve constraints of Figure 2 and gathered experimental robot data for five constraints. For the simulation experiments we used prior knowledge of the constraint frame types $\left\{\phi \right\}$ and $\left\{\psi \right\}$ , to test how identification accuracy is affected by noise added to the poses $T\left(k\right)$ , and consequently twists $V\left(k\right)$ . For the robot experiments we used no prior knowledge of the frame type. We classified the constraint frame types and identified the constraint geometry, with real-life noise.

### 5.1 Simulation experiments

For the simulation experiments, we first generated constrained end effector poses $T\left(k\right)$ using Python 3.11 and NumPy 1.26. The velocities were $\omega^{\phi }\left(k\right)=d^{\phi }\circ 0.5\circ {\left[1+\sin\left(2k\Delta t\right),-1,\cos\left(3k\Delta t\right)\right]}^{T}\text{ rad }s^{-1}$ and $v^{\psi }\left(k\right)=d^{\psi }\circ 0.1\circ {\left[-1,\cos\left(2k\Delta t\right),\sin\left(3k\Delta t\right)\right]}^{T}\;m\;s^{-1}$ where ∘ is element-wise multiplication and Δt is the sample time. Therefore, if $d^{\phi }$ and $d^{\psi }$ specify that an axis is constrained, the velocity along that axis will be zero. Different $\left\{\phi \right\}$ and $\left\{\psi \right\}$ then result in all constraints of Figure 2. The end effector twist $V^{b}\left(k\right)$ was calculated using the transformation of Equation 2.

Poses $T\left(k\right)$ were obtained using the discrete-time version of Equation 1 for constant $V^{b}\left(k\right)$ between samples (Lynch and Park, 2017):

$$T\left(k+1\right)=\exp\left({\left[V^{b}\left(k\right)\right]}_{\times }\Delta t\right)\;T\left(k\right).$$ (9)

**FIGURE 3:**
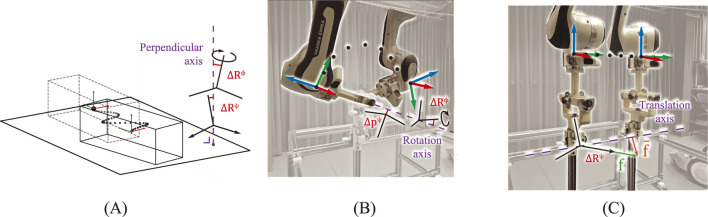
**(A)** Constraint frame identification of a plane-plane constraint (Figure 2D) in simulation experiments. **(B)** Constraint frame identification of a revolute joint (Figure 2A) in robot experiments. **(C)** Reproduction of motion in a prismatic joint (Figure 2B) by applying force f along the identified free axis of the constraint frame containing error $\Delta R^{\psi }$ , the reaction forces $\bar{f}$ are due to the real-world constraint. The setup in **(B,C)** can be configured to constrain specific axes. Measured end effector poses are shown as black dots with red-green-blue axes. Errors in the identified constraint frame geometry are shown enlarged in red with respect to the purple ground-truth geometry: perpendicular axis in **(A)**, rotation axis in **(B)**, and translation axis in **(C)**.

Discrete time ran for 5 s with sample time Δt=0.1 s , an example is shown in Figure 3A. To mimic real world experiments, we introduced noise to $T\left(k\right)$ . Normally distributed noise with standard deviation $\sigma_{p}$ was added to the position $p\left(k\right)$ in $T\left(k\right)$ .

Normally distributed noise with standard deviation $\sigma_{R}$ was added to the rotation vector representation $\theta \left(k\right)\in R^{3}$ of $R\left(k\right)$ in $T\left(k\right)$ , related by ${\left[\theta \left(k\right)\right]}_{\times }=\ln\left(R\left(k\right)\right)$ . We assumed that the noise on position $\sigma_{p}$ and rotation $\sigma_{R}$ components were the result of one noise source with standard deviation σ , such that $\sigma_{p}=\sigma$ and $\sigma_{R}=3\sigma \;m^{-1}$ . Therefore, varying σ affects both position and rotation components in the noisy poses $\tilde{T}\left(k\right)$ . The three-times larger effect of σ on $\sigma_{R}$ was estimated by measuring noise on the robot pose in standstill.

### 5.2 Robot experiments

To test the method on real-world data, a Franka Research 3 (Franka Robotics, Munich, Germany) was used to collect constrained end effector poses $\tilde{T}\left(k\right)$ , an example is shown in Figure 3B. The experimenter physically moved the end effector through space in low-impedance guiding mode, which is part of the robot’s default control options, while the end effector was bolted to a constrained mechanical setup. While interacting with a user in the guiding mode, the robot complied with the Franka safety considerations. These consist of limits on all motor positions, velocities, accelerations, jerks, torques, and torque derivatives. The robot stops upon violation of these limits.

Poses $\tilde{T}\left(k\right)$ of five constraints (Figures 2A, B, D, I, K) were collected over ten trials per constraint for 5 s at Δt=0.05 s . The experimenter attempted to track the poses of the simulations.

### 5.3 Error metrics for evaluation

Section 3.3 noted that the geometric parameters $R^{\phi }$ , $R^{\psi }$ , and $p^{\psi }$ may contain redundancies depending on the constraints. Therefore, the error metrics reflect this.

Section 3.3 and Table 2 noted that $R^{\phi }$ and $R^{\psi }$ either have none or one unique basis vector in $R^{\phi }$ and none or one in $R^{\psi }$ . If there are no unique basis vectors, the error is irrelevant. If there is one unique basis vector l , the error metric is the angle $\Delta R=\mathrm{acos}\left(r_{l}\cdot {\hat{r}}_{l}\right)\in R$ between the ground-truth basis vector $r_{l}$ and the estimated basis vector ${\hat{r}}_{l}$ . Angle ΔR was wrapped at ±90 deg , as the positive and negative axes are irrelevant. For the error metric of $p^{\psi }$ , the Euclidian distance from the ground truth $\Delta p^{\psi }={\left\|p^{\psi }-{\hat{p}}^{\psi }\right\|}_{2}$ was used. Only the unique components of $p^{\psi }-{\hat{p}}^{\psi }$ , as shown in Table 2, were used.

The ground truth constraint frame geometries were $R^{\phi }=R^{\psi }=I_{3\times 3}$ , with $p^{\psi }={\left[0.1,0.3,0.1\right]}^{T}\;m$ for simulation, and $p^{\psi }={\left[0.0,0.0,0.244\right]}^{T}\;m$ for robot experiments.

### 5.4 Implementation

Given experimental poses $\tilde{T}\left(k\right)$ , noisy twists ${\tilde{V}}^{b}\left(k\right)$ were computed from Equation 9 by solving for $V^{b}\left(k\right)$ . The optimizations of Equation 7 and Equation 8 were implemented in Python using NumPy and the SciPy 1.10 dual annealing global optimization algorithm with 100 maximum iterations and default settings otherwise (Xiang et al., 2013). Our code is available online^1^.

The rotation matrices $R^{\phi }$ , $R^{\psi }$ were parameterized by their rotation vector representations and bounded to ±180 deg . The bounds on $p^{\psi }$ were ±1 m . Initial guesses were randomized. For the simulation data, optimizations were done with added noise $\sigma \in \left[10^{-6},10^{-4}\right]\;m$ spaced exponentially at 10 values. For the robot data, optimizations were done with no added noise σ . One optimization of Equation 7 or Equation 8 for a single frame type took approximately 2 s on an Intel Core i5-6600 CPU with 8 GB RAM.

### 5.5 Simulation experiment results

**FIGURE 4:**
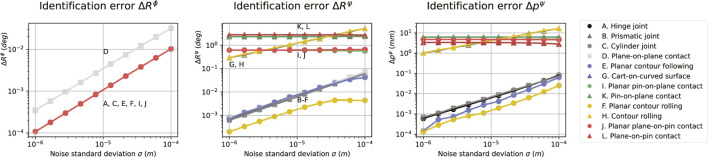
Sensitivities of the geometric identification errors ( $\Delta R^{\phi }$ , $\Delta R^{\psi }$ , $\Delta p^{\psi }$ ) to noise ( σ ) in simulation experiments for all constraints of Figure 2.

The geometric errors ( $\Delta R^{\phi }$ , $\Delta R^{\psi }$ , $\Delta p^{\psi }$ ) relate to noise ( σ ) with approximately unit slope (Figure 4) for all constraints except for I, J, K, and L, whose errors ( $\Delta R^{\psi }$ , $\Delta p^{\psi }$ ) are constant with increasing noise. Constraints with fewer degrees of freedom are generally identified more accurately.

### 5.6 Robot experiment results

Identifying constraint frames in demonstrations resulted in several candidate frame types (left, Table 4), each having associated degrees of freedom (middle, Table 4) and geometry (right, Table 4).

**TABLE 4:** Identified constraint frames of robot demonstrations.

Figure 2 label	Min. norm $\left(7\right)\text{ in frame type }\left\{\phi \right\}\;\left(\text{mrad }s^{-1}\right)$				Degrees of freedom*			Geometry error*	
	Min. norm $\left(8\right)\text{ in frame type }\left\{\psi \right\}\;\left(\text{mm }s^{-1}\right)$								
	$\left\{G\right\}$		$\left\{B\right\}$		$\lambda^{\phi }\;\left(\text{mrad }s^{-1}\right)$			${\Delta R}^{\phi }\;\left(\deg\right)$	−
	$\left\{BB\right\}$	$\left\{GB\right\}$	$\left\{GG\right\}$	$\left\{BG\right\}$	$\lambda^{\psi }\;\left(\text{mm }s^{-1}\right)$			${\Delta R}^{\psi }\;\left(\deg\right)$	$\Delta p^{\psi }\left(\text{mm}\right)$
A	108.2±7.9		20.5±10.8		37.9±2.8	47.2±3.8	*863.7* ± 46 .7	0.19±0.02 ^‡^	−
	2.4±0.2	3.6±0.2	2.3±0.1	2.3±0.2	1.6±0.2	1.8±0.2	4.5±0.2	−	6.46±0.21
B	47.5±3.7		47.5±3.7		39.0±3.5	50.0±3.8	55.3±4.4	−	−
	12.9±1.1	7.9±0.5	21.3±1.5	23.3±1.6	0.9±0.1	1.8±0.2	*256.5* ± 13.4	0.94±0.18 ^‡^	−
D	144.3±10.1		142.1±9.2		50.5±3.7	60.8±3.5	*958.2* ± 115.6	2.59±0.50	−
	47.9±7.2	35.1±2.3	60.3±7.2	58.4±8.1	1.4±0.2	*156.4* ± 25.3	*185.7* ± 14.2	0.52±0.13	−
I	85.4±5.0		86.3±2.3		25.5±1.7	30.3±3.9	*786.5* ± 77.5	0.56±0.15	−
	18.0±1.9	10.6±1.2	23.1±2.3	23.5±2.1	2.4±0.3	5.2±0.9	*93.9* ± 7.3	1.64±0.18	3.53±1.12 ^‡^
K	428.3±2.1		495.3±4.0		*260.2* ± 30.3	*430.8* ± 78.8	*716.1* ± 71.6	−	−
	54.2±10.8	30.3±4.9	56.4±8.1	59.8±11.0	4.2±1.4	*68.3* ± 17.8	*99.4* ± 14.6	2.45±1.43	7.69±2.32

**FIGURE 5:**
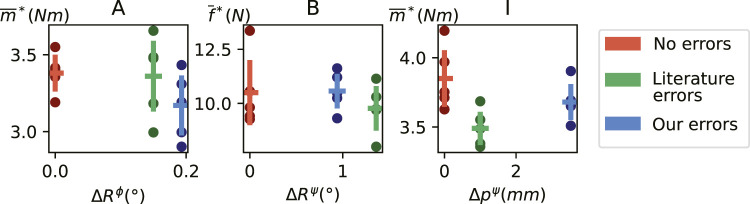
The peak magnitude reaction wrenches ( ${\bar{f}}^{*}$ , ${\bar{m}}^{*}$ ) and their means and standard deviations over five reproductions of the tasks containing constraints **(A–C)**. Colors represent the source of the errors in the constraint frame used in the control.

* In frame type with lowest minimized norm (**bold**). *Italic*: corresponds to ground-truth free axis. ^‡^Error used in reproduction (Figure 5).

Section 4 noted that optimal frame types may be classified as the one with the lowest norms of Equation 7 or Equation 8. For constraints where any frame type is valid, norms are expected to be similar between frame types. This holds for all $\left\{\phi \right\}$ and $\left\{\psi \right\}$ in A, but not for $\left\{\psi \right\}$ in B, D which have lowest norm in $\left\{GB\right\}$ . For constraints where only one frame type is valid ( $\left\{GB\right\}$ in I, K) the norms of the ground truth types are approximately half those of the other types. Therefore, frame types were classified correctly by the frame with the lowest minimized norm.

After classifying the optimal frame type, the associated degrees of freedom and geometry can be evaluated (middle, right, Table 4). If any threshold between $\left[60,260\right]\text{ mrad }s^{-1}$ is applied to all $\lambda^{\phi }$ , and any between $\left[6,68\right]\text{ mm }s^{-1}$ to all $\lambda^{\psi }$ , the degrees of freedom of all five constraints (Table 2) are classified correctly. Suitable thresholds may be chosen at the midpoint of these ranges: $160\text{ mrad }s^{-1}$ for $\lambda^{\phi }$ and 37 $\text{mm }s^{-1}$ for $\lambda^{\psi }$ . These midpoint thresholds are at least 5 standard deviations away from the means of $\lambda^{\phi }$ and $\lambda^{\psi }$ . The errors of the identified geometry are within $\Delta R^{\phi }<1\;\deg$ , $\Delta R^{\psi }<2.5\;\deg$ and $\Delta p^{\psi }<8\text{ mm}$ .

## 6 Application to robot task reproduction

This section illustrates how robot tasks can be reproduced using control expressed in the identified constraint frames of Section 4, and how their identification errors ( $\Delta R^{\phi }$ , $\Delta R^{\psi }$ , $\Delta p^{\psi }$ ) may influence task performance. The Franka robot reproduced three straightforward endstop-to-endstop tasks involving constraints A, B, and I enforced by the mechanical setup from Section 5.2. An experiment is visualized in Figure 3C. We collected five trials for each task at a 0.05 s sample time and clipped each trial to 5 s .

### 6.1 Reproduction control

To reproduce the tasks, we used simple control based on the identified constraint frames from the experiments of Table 4, thereby illustrating simple LfD. We sent desired motor torques $\tau \in R^{7}$ to the Franka torque controller based on constant desired wrenches (moments $m\in R^{3}$ and forces $f\in R^{3}$ ) expressed in the identified constraint frames. The torques and wrenches are related by $\tau =J^{T}{\left[f^{T}\;m^{T}\right]}^{T}$ , where $J\in R^{6\times 7}$ is the robot pose-dependent Jacobian (Lynch and Park, 2017).

Desired motor torques were sent to the robot using Robotic Operating System (ROS, version Noetic Ninjemys) from Ubuntu 20.04 with the *franka_ros* package. During operation, the user and bystanders were outside the workspace of the robot, and the user monitored the task reproduction with the robot’s emergency stop in hand.

Desired wrenches were set to constant values ( 3.5 Nm and 7 N ) along the free axes of the identified constraint frames, and to zero ( 0 Nm and 0 N ) along the constrained axes. Therefore, the control for constrained task A applied a pure moment using the identified $R^{\phi }$ . The control for constrained task B applied a pure force using the identified $R^{\psi }$ . The control for constrained task I applied a moment and a force using the identified $R^{\phi }$ , $R^{\psi }$ , and $p^{\psi }$ , but for this experiment we set $\Delta R^{\phi }=\Delta R^{\psi }=0$ to isolate the effect of $\Delta p^{\psi }$ .

### 6.2 Effects of identification accuracy

During constrained task reproduction there will be reaction wrenches along the constrained axes, which we consider undesirable in these experiments. Such reaction wrenches can be caused by, among other factors, the control applying wrenches based on imperfect constraint identification. Therefore, we compared the reaction wrenches in case the constraint frames used in the controller contain no errors ( $\Delta R^{\phi }=\Delta R^{\psi }=\Delta p^{\psi }=0$ ), identification errors from methods in the literature that use prior knowledge (De Schutter et al., 1999; Niekum et al., 2015), and identification errors from our method without prior knowledge of the constraint (Table 4).

From the measured interaction wrenches ( $m\left(k\right)$ and $f\left(k\right)$ ) we determined the reaction wrenches (moments $\bar{m}\left(k\right)$ and forces $\bar{f}\left(k\right)$ ) along the ground-truth constrained axes (Figure 2), and determined their peak magnitudes ${\bar{m}}^{*}$ and ${\bar{f}}^{*}$ with ${\bar{m}}^{*}=\max{\left\|\bar{m}\left(k\right)\right\|}_{2}\in R$ . Thereby, we obtain a metric of how constraint identification accuracy influences undesirable reaction wrenches in task reproduction. The results are shown in Figure 5. Larger identification errors (i.e., from our method) were expected to cause larger reaction wrenches, but this is not necessarily the case. In all reproductions, the physical endstops of the setup were reached.

## 7 Discussion and conclusion

This work identifies constraints from kinematic data using *constraint frames*, consisting of a frame *type* that determines what body the frame is attached to, *geometry* that determines the orientation and position, and the *degrees of freedom* of velocities in that frame. First, frame geometries are identified by minimizing a norm on velocities in all frame types. Second, the optimal frame type is classified as the one with the lowest norm. Third, the degrees of freedom can be classified by thresholding the velocities in that frame.

### 7.1 Advantages

The method does not require force measurements, and can therefore be applied to any system that measures positions and orientations of constrained objects or manipulators. Examples include other (mobile) robotic arms with different kinematic chains, humanoid robots, and mobile robots constrained by their environments. Besides robotics, the method may also be applied to motion tracking systems, for example, to monitor human movement or estimate human joints (Ancillao et al., 2022). While impedance control was used to collect pose data and reproduce tasks, the constraint identification itself does not depend on force measurements. Because force measurements can improve identification accuracy it is the topic of future work (De Schutter et al., 1999; Subramani et al., 2020). Moreover, correct kinematics-based constraint identification requires that all true degrees of freedom are excited. Otherwise, there is no discernible difference between, e.g., a prismatic joint constraint and unconstrained but straight line translation, which will have the same apparent degrees of freedom.

The method requires no specific prior constraint models, nor estimates of geometry. Hence, there is no need to manually estimate or define constraints, which may be challenging to do exhaustively for all constraints that can be encountered in everyday settings. The method can identify a wide variety of constraints, of which twelve common examples were shown. However, many more can be identified by enumerating over all possibilities, including constraints where the angular and linear velocity frames are not orthogonal.

The method identifies a fixed number of intuitive parameters (Table 1). Methods that fit specific constraint models must identify and choose from multiple candidate models, each with their own parameters. Therefore, our method may be more efficient when many different constraints must be considered, such as in everyday settings.

Our identified constraint frames can be directly interpreted as “task frames” from the literature since both frames conveniently describe contact tasks (Bruyninckx and De Schutter, 1996; Mousavi Mohammadi et al., 2024). Although recent work on task frame identification by Mousavi Mohammadi et al. (2024) also has the above advantages, in this work we also classify constraints by discerning the degrees of freedom associated with our frames. Although we reproduced simple tasks, such frames can also be used to reproduce more complicated contact tasks with hybrid position and force control applied to the task frame axes (Conkey and Hermans, 2019; Ureche et al., 2015). Alternatively, impedance controller stiffness may be varied based on the identified constraints.

### 7.2 Limitations and future work

In this work, we applied our method to a window of kinematic data which we assume contains a single constraint. In future work, we will investigate how our method can be applied to data containing sequential constraints. Furthermore, we only considered single-contact constraints which can be modeled using a single constraint frame. Multiple-contact constraints, such as the two ends of a stick contacting separate planes, cannot be fully modeled using a single instance of the current method, since only one of the two contact points will be identified. However, a second instance of our method may identify a second contact point, if the second solution is (forced to be) distinct. Therefore, multiple contacts may be identified with multiple instances of our method, which is the topic of future work.

We defined constraint identification as a minimization problem and applied a general global-local optimization method. Improvements in accuracy and time efficiency may be achieved in several ways: by more efficient optimizer implementations, by choosing a different optimization method suited for our specific problem, or by tuning the optimization parameters. Furthermore, prior information may be useful for initialization. For example, a vision system may be used to identify a tool tip as prior information on constraint geometry.

Identifying constraint geometry does not require any parameter choice, but classifying the degrees of freedom requires two thresholds on RMS velocities, which can be chosen heuristically or empirically. Heuristically, thresholds may be chosen based on prior information, such as expected noise and expected velocities, which may differ between applications and measurement systems. If such prior information is available, classification may perform as expected without the need for threshold tuning. Empirically, thresholds may be chosen following demonstrations with known ground-truth constraints (Section 5). If such demonstrations are available and representative of future tasks, no explicit prior information about noise and velocities are needed, which may be more convenient depending on the application.

Geometric identification errors were found to scale linearly with noise in simulation experiments, and the exact scaling varies with the underlying constraints. Errors in robot experiments were larger than those in simulation experiments, which may be due to unmodeled factors such as structural compliance. Classification of the ground-truth constraint frame type was successful in all robot experiments. An analysis of factors that influence identification and classification performance is outside the scope of this work, such as the constraint, the optimizer, the relative direction and magnitude of motion, and the sample size.

Constraint identification methods in the literature report geometric identification accuracies using different metrics. In our method, we report the geometric parameter errors between points, lines, and planes. Methods that use similar metrics report similar errors: within 1–7.5 mm and 0.5–5 deg for point-on-plane contacts, prismatic, and revolute joints (De Schutter et al., 1999; Sturm et al., 2011; Mousavi Mohammadi et al., 2024). Other methods report the fitness between observations and their candidate constraint models (Sturm et al., 2011; Subramani et al., 2018; Subramani et al., 2020; van der Walt et al., 2025). Of such methods, the most accurate report sub-millimeter mean fit errors (Subramani et al., 2018). While such sub-millimeter mean model fit errors are not directly comparable to geometric parameter errors, they may correspond to sub-millimeter geometric errors.

While our method may be more versatile, it may yield larger geometric identification errors than methods that fit specific constraint models to data. Regardless, such larger errors (e.g., 5 mm instead of 0.5 mm) did not lead to substantially larger reaction wrenches in simple reproduction experiments with the default Franka controller (Section 6). Furthermore, similar errors have been shown to lead to acceptable performance in more complicated tasks in related work (Mousavi Mohammadi et al., 2024). The obtained accuracy may therefore be sufficient for such tasks, and other factors may have a greater effect on task performance, such as the task definition, environment, robot, controller, and definition of success. For example, control methods that are designed for compliant contact, such as impedance control, may be more robust to misidentified constraint geometry than non-compliant control. We aim to apply such compliant control methods to reproduce constrained tasks through LfD in future work.

### 7.3 Conclusion

This work identified constraint frames in robot tasks without prior knowledge of the constraints or tasks and without force measurements. Automatically modeling such robot-environment interactions, for example, in the context of Learning from Demonstration, may support versatile autonomous robot applications.

## Data Availability

The raw data supporting the conclusions of this article will be made available by the authors, without undue reservation.

## Footnotes

1
https://github.com/ET-BE/ReFrameId

## References

[B1] Ancillao A. Verduyn A. Vochten M. Aertbeliën E. De Schutter J. (2022). A novel procedure for knee flexion angle estimation based on functionally defined coordinate systems and independent of the marker landmarks. Int. J. Environ. Res. Public Health 20, 500. 10.3390/ijerph20010500 36612839 PMC9819753

[B2] Bruyninckx H. De Schutter J. (1993a). Kinematic models of rigid body interactions for compliant motion tasks in the presence of uncertainties. Proc. IEEE Int. Conf. Robotics Automation 1, 1007–1012. 10.1109/ROBOT.1993.292107

[B3] Bruyninckx H. De Schutter J. (1996). Specification of force-controlled actions in the “task frame formalism”-a synthesis. IEEE Trans. robotics automation 12, 581–589. 10.1109/70.508440

[B4] Bruyninckx H. De Schutter J. Dutre S. (1993b). The “reciprocity” and “consistency” based approaches to uncertainty identification for compliant motions. Proc. IEEE Int. Conf. Robotics Automation 1, 349–354. 10.1109/ROBOT.1993.292006

[B5] Bullen P. S. (2003). Handbook of means and their inequalities. Netherlands, Dordrecht: Springer.

[B6] Cabras S. Castellanos M. E. Staffetti E. (2010). Contact-state classification in human-demonstrated robot compliant motion tasks using the boosting algorithm. IEEE Trans. Syst. Man, Cybern. Part B Cybern. 40, 1372–1386. 10.1109/TSMCB.2009.2038492 20106744

[B7] Conkey A. Hermans T. (2019). Learning task constraints from demonstration for hybrid force/position control. IEEE-RAS Int. Conf. Humanoid Robots 2019-Octob, 162–169. 10.1109/Humanoids43949.2019.9035013

[B8] de Schutter J. Bruyninckx H. Dutré S. de Geeter J. Katupitiya J. Demey S. (1999). Estimating first-order geometric parameters and monitoring contact transitions during force-controlled compliant motion. Int. J. Robotics Res. 18, 1161–1184. 10.1177/02783649922067780

[B9] Hausman K. Niekum S. Osentoski S. Sukhatme G. S. (2015). “Active articulation model estimation through interactive perception,” in 2015 IEEE international conference on robotics and automation (ICRA). Presented at the 2015 (IEEE International Conference on Robotics and Automation ICRA), 3305–3312. 10.1109/ICRA.2015.7139655

[B10] Holladay R. Lozano-Pérez T. Rodriguez A. (2021). Planning for multi-stage forceful manipulation. arXiv, 6556–6562. 10.1109/icra48506.2021.9561233

[B11] Jain A. Niekum S. (2018). “Efficient hierarchical robot motion planning under uncertainty and hybrid dynamics,” in Proceedings of the 2nd conference on robot learning. Presented at the conference on robot learning (PMLR), 757–766.

[B12] Kroemer O. Niekum S. Konidaris G., (2020). A review of robot learning for manipulation: challenges, representations, and algorithms. arXiv:1907.03146.

[B13] Lefebvre T. Bruyninckx H. Schutter J. D. (2005). Online statistical model recognition and State estimation for autonomous compliant motion. IEEE Trans. Syst. Man, Cybern. Part C Appl. Rev. 35, 16–29. 10.1109/TSMCC.2004.840053

[B14] Li X. Baum M. Brock O. (2023). “Augmentation enables one-shot generalization in learning from demonstration for contact-rich manipulation,” in 2023 IEEE/RSJ international conference on intelligent robots and systems (IROS). Presented at the 2023 (IEEE/RSJ International Conference on Intelligent Robots and Systems IROS), 3656–3663. 10.1109/IROS55552.2023.10341625

[B15] Li X. Brock O. (2022). Learning from demonstration based on environmental constraints. IEEE Robotics Automation Lett. 7, 10938–10945. 10.1109/LRA.2022.3196096

[B16] Lynch K. M. Park F. C. (2017). Modern robotics: mechanics, planning, and control. Cambridge University Press.

[B17] Meeussen W. Rutgeerts J. Gadeyne K. Bruyninckx H. De Schutter J. (2007). Contact-state segmentation using particle filters for programming by human demonstration in compliant-motion tasks. IEEE Trans. Robotics 23, 218–231. 10.1109/tro.2007.892227

[B18] Mousavi Mohammadi S. A. Vochten M. Aertbeliën E. De Schutter J. (2024). Automatic extraction of a task frame from human demonstrations for controlling robotic contact tasks. arXiv. 10.48550/arXiv.2404.01900

[B19] Niekum S. Osentoski S. Atkeson C. G. Barto A. G. (2015). Online Bayesian changepoint detection for articulated motion models. Proc. - IEEE Int. Conf. Robotics Automation 2015-June, 1468–1475. 10.1109/ICRA.2015.7139383

[B20] Oriolo G. Vendittelli M. (2009). “A control-based approach to task-constrained motion planning,” in 2009 IEEE/RSJ international conference on intelligent robots and systems. Presented at the 2009 IEEE/RSJ international conference on intelligent robots and systems, 297–302. 10.1109/IROS.2009.5354287

[B21] Overbeek A. H. G. Dresscher D. Van der Kooij H. Vlutters M. (2025). Versatile kinematics-based constraint identification applied to robot task reproduction. Preprint. Available online at: https://research.utwente.nl/en/publications/versatile-kinematics-based-constraint-identification-applied-to-r (Accessed May 31, 2025). 10.3389/frobt.2025.1574110 PMC12303821 40735746

[B22] Simonič M. Ude A. Nemec B. (2024). Hierarchical learning of robotic contact policies. Robotics Computer-Integrated Manuf. 86, 102657. 10.1016/j.rcim.2023.102657

[B23] Slaets P. Lefebvre T. Rutgeerts J. Bruyninckx H. De Schutter J. (2007). Incremental building of a polyhedral feature model for programming by human demonstration of force-controlled tasks. IEEE Trans. Robotics 23, 20–33. 10.1109/TRO.2006.886830

[B24] Sturm J. Jain A. Stachniss C. Kemp C. C. Burgard W. (2010). “Operating articulated objects based on experience,” in 2010 IEEE/RSJ international conference on intelligent robots and systems. Presented at the 2010 IEEE/RSJ international conference on intelligent robots and systems, Taipei, Taiwan, 2739–2744. 10.1109/IROS.2010.5653813

[B25] Sturm J. Stachniss C. Burgard W. (2011). A probabilistic framework for learning kinematic models of articulated objects. J. Artif. Intell. Res. 41, 477–526. 10.1613/jair.3229

[B26] Subramani G. Hagenow M. Gleicher M. Zinn M. (2020). A method for constraint inference using pose and wrench measurements. arXiv. 10.48550/arXiv.2010.15916

[B27] Subramani G. Zinn M. Gleicher M. (2018). “Inferring geometric constraints in human demonstrations,” in Proceedings of the 2nd conference on robot learning. Presented at the conference on robot learning (Zürich, Switzerland: PMLR), 223–236.

[B28] Suomalainen M. Abu-Dakka F. J. Kyrki V. (2021). Imitation learning-based framework for learning 6-D linear compliant motions. Auton. Robot. 45, 389–405. 10.1007/s10514-021-09971-y

[B29] Ureche A. L. P. Umezawa K. Nakamura Y. Billard A. (2015). Task parameterization using continuous constraints extracted from human demonstrations. IEEE Trans. Robotics 31, 1458–1471. 10.1109/TRO.2015.2495003

[B30] Van der Walt C. Stramigioli S. Dresscher D. (2025). Mechanical constraint identification in model-mediated teleoperation. IEEE Robotics Automation Lett. 10, 5777–5782. 10.1109/LRA.2025.3560893

[B31] Xiang Y. Gubian S. Suomela B. Hoeng J. (2013). Generalized simulated annealing for global optimization: the GenSA package. R J. 5 (1), 13–29. 10.32614/RJ-2013-002

